# The p53 Family Members p63 and p73 Roles in the Metastatic Dissemination: Interactions with microRNAs and TGFβ Pathway

**DOI:** 10.3390/cancers14235948

**Published:** 2022-12-01

**Authors:** Lidia Rodriguez Calleja, Melanie Lavaud, Robel Tesfaye, Bénédicte Brounais-Le-Royer, Marc Baud’huin, Steven Georges, François Lamoureux, Franck Verrecchia, Benjamin Ory

**Affiliations:** Nantes Université, CHILD Team, CRCI^2^NA, INSERM UMR 1307, CNRS UMR 6075, F-44000 Nantes, France

**Keywords:** p53, TGF, metastasis

## Abstract

**Simple Summary:**

The p53 protein family is a class of proteins successively known to be the guardians of the genome, but also depending on the different isoforms have pro-tumoral and pro-metastatic potential. This dual potential is also observed within the TGFb pathway. Several interactions between those two proteins family start to explain this complexity.

**Abstract:**

TP53 (TP53), p73 (TP73), and p63 (TP63) are members of the p53 transcription factor family, which has many activities spanning from embryonic development through to tumor suppression. The utilization of two promoters and alternative mRNA splicing has been shown to yield numerous isoforms in p53, p63, and p73. TAp73 is thought to mediate apoptosis as a result of nuclear accumulation following chemotherapy-induced DNA damage, according to a number of studies. Overexpression of the nuclear ΔNp63 and ΔNp73 isoforms, on the other hand, suppresses TAp73’s pro-apoptotic activity in human malignancies, potentially leading to metastatic spread or inhibition. Another well-known pathway that has been associated to metastatic spread is the TGF pathway. TGFs are a family of structurally related polypeptide growth factors that regulate a variety of cellular functions including cell proliferation, lineage determination, differentiation, motility, adhesion, and cell death, making them significant players in development, homeostasis, and wound repair. Various studies have already identified several interactions between the p53 protein family and the TGFb pathway in the context of tumor growth and metastatic spread, beginning to shed light on this enigmatic intricacy.

## 1. p53 Protein Family

The p53 transcription factor family, which includes TP53 (TP53), p73 (TP73), and p63 (TP63), is a protein family that has a wide range of functions, ranging from embryonic development through to tumor suppression [1]. Unlike p53, p63 and p73 knockout (KO) mice showed abnormal epithelial development, with truncated limbs, missing lachrymal or salivary glands, and missing teeth and hair follicles [2,3]. p63 and p73 knockout (KO) mice also showed defective neurological development, with congenital hydrocephalus, hippocampal dysgenesis, and chronic inflammation, in the case of p73 KO [4,5].

In response to various cellular stress conditions such as DNA damage, hypoxia, nucleotide imbalance, and others, the tumor suppressor p53 induces target genes that are involved in cell cycle arrest, apoptosis, and DNA repair [6]. Most human cancers have p53 inactivation due to direct mutation, deletion, or disruption of critical regulatory mechanisms that are required for proper p53 function. Mutations or deletion of the p63 and p73 transcription factors, on the other hand, are uncommon in cancer [7,8].

### p53 Protein Family: Structure and Functions

**Figure 1:**
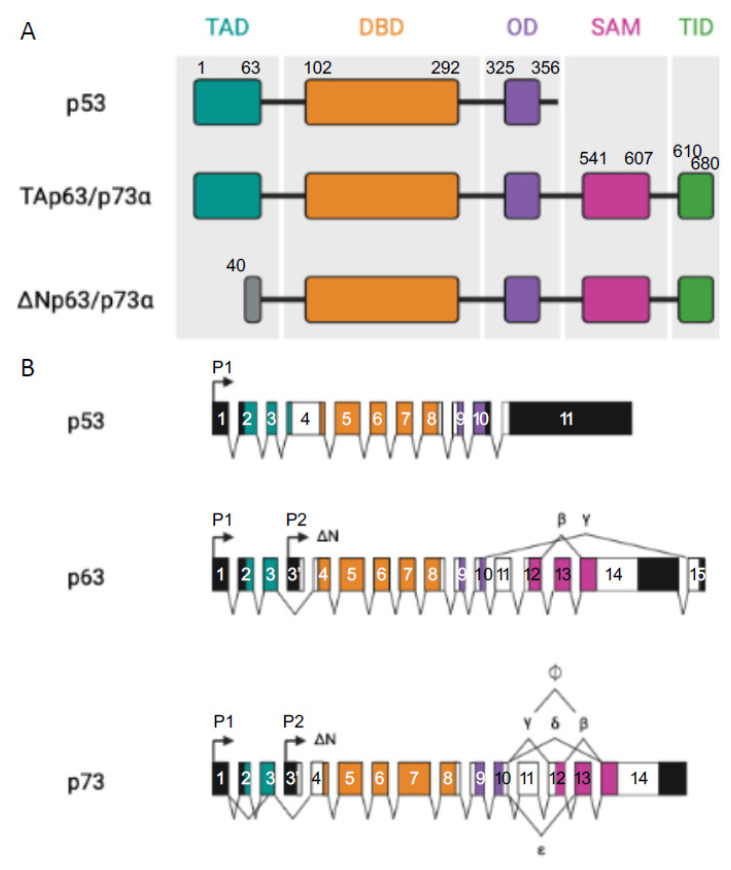
p53 family members. (**A**) Representation of the different domains in p53 and TA and ΔN p63α and p73α isoforms: transactivation domain (TA, in light grey), DNA binding domain (DBD, in red), oligomerization domain (OD, in yellow), sterile alpha domain (SAM, in dark grey), and the transactivation inhibitory domain (TID, in orange), introns (black lines). (**B**) Represents p53, p63, and p73 multiple spliced variants. P stands for different promoters, numbered boxes indicate exons, black boxes are untranslated sequences, and black lines are introns.

All three p53 family members are very similar and present a high homology both at the genomic and protein levels. Each contains an N-terminal transactivation domain (TAD), a central DNA binding domain (DBD) through which they regulate both shared and distinct transcriptional targets [9], and an oligomerization domain (OD) [1]. In addition, p63 and p73, but not p53, can contain a long C-terminal that is mainly composed of a sterile alpha motif (SAM) domain and a transactivation inhibitory domain (TID). The SAM consists of four α-helices and a small helix which enables protein–protein interactions whereas TID is a region that inhibits the transcriptional activity of TA isoforms through inter- or intra-molecular association with their TAD [10,11] (Figure 1). The DBD exhibits more than 60% homology between the three proteins, suggesting that they can bind to similar sequences and transactivate the same promoters. Furthermore, the high conservation of OD suggests the possibility of homo- and hetero-oligomer formation between the p53 family protein partners.

Moreover, p63 and p73 can be expressed from two distinct promoters (one upstream of exon 1 (P1) and another that is located within intron 3 (P2)) and can also be differentially spliced, thereby producing different isoforms. Transcription from the P1 promoter gives an N-terminal acidic TA domain (TAp63 and TAp73) whereas in the products that are transcribed from P2, this TA domain is absent (ΔNp63 and ΔNp73) [12].

From the C-terminal splicing of p63 and p73, a large variety of proteins can be generated. At least seven C-terminal isotypes have been identified for p73 (α, β, γ, δ, ε, ζ, and η) and three for p63 (α, β, and γ). In total, the p63 gene encodes for six different protein isoforms and the p73 gene expresses 35 mRNA variants that can theoretically encode for 28 different protein isoforms. Only 14 isoforms have been described so far [13]. Their contributions are not yet fully understood but some evidence points to the fact that these different C-termini could be involved in the capacity of TA isoforms to transactivate gene expression [7,8]. In both cases, p63α and p73α are the full-length isoforms (with SAM and TID present at their C-termini) and the others are the result of different truncations of those ones (Figure 1).

It has been shown that p53 also produces multiple isoforms through the use of two promoters and alternative mRNA splicing. Those isoforms are expressed in normal human tissues in a tissue-dependent manner [13].

Due to the high homology that is observed within the three members of the p53 protein, and the fact that, unlike the not functional p53, p63 and p73 are found overexpressed in the vast majority of cancers and their role in the malignant context has been questioned [14]. Whether p63 and p73 promote or not, human tumorigenesis and metastatic dissemination may depend on the predominant isoforms that are expressed in a specific tissue. TA isoforms have been demonstrated to transactivate distinct but overlapping subsets of known p53-regulated genes that are involved in cell-cycle arrest and apoptosis, as well as some other gene sets not regulated by p53. In contrast, the TAD truncated isoforms, ΔNp63 and ΔNp73 proteins, function as dominant-negative inhibitors of the p53 family, so that, these isoforms may be most likely related with protooncogenic functions [15,16]. Those conclusions come from studies indicating that TAp73 mediates apoptosis as a result of its nuclear accumulation following chemotherapy-induced DNA damage [17]. The overexpression of ΔNp63 and ΔNp73, in contrast, inhibits the pro-apoptotic effect of Tap73 in human tumors.

## 2. p63 and p73 Interactions in the Head and Neck Squamous Cell Carcinoma Model (HNSCC)

Head and neck squamous cell carcinoma (HNSCC) is the most common type (90%) of head and neck cancers, a group of biologically similar malignancies that affects the oral cavity (mouth), nasal cavity, pharynx, larynx, and paranasal sinuses. It arises from epithelial cells that line the mucosal surfaces of the head and the neck [18]. HNSCC is the sixth most common diagnosed cancer worldwide with 560,000 new cases and 300,000 deaths annually reported [19,20]. Although cervical lymph nodes are the main metastatic sites, the risk of dissemination is not so high and it depends on both the stage and location of the primary tumor [21]. Nevertheless, head and neck cancers are aggressive in nature. Tobacco and alcohol consumption are the main factors that are responsible for HNSCC apparition but some studies also point their initiation from the human papillomavirus (HPV) infection. Therapy is mainly based on surgery or radiotherapy at early stages whereas a combination of surgery, radiotherapy, and chemotherapy is applied in advanced stages, resulting in multiple toxic side effects [18].

**Figure 2:**
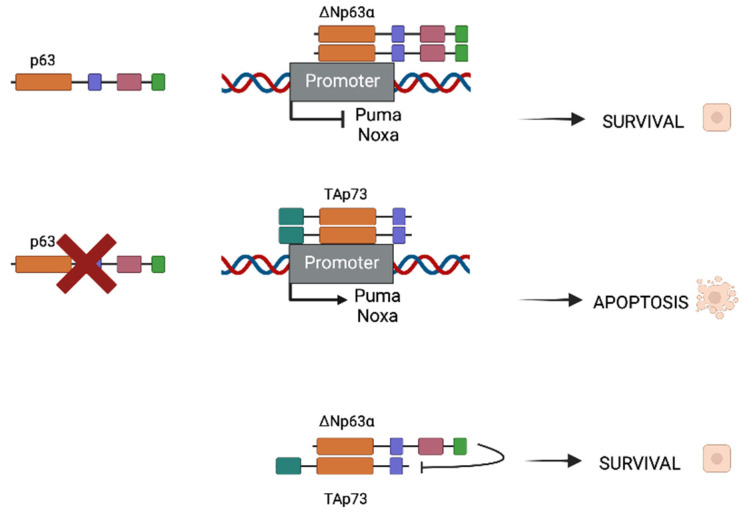
Different oligomerization of ΔNp63α and TAp73 depending on p63 presence or absence. When ΔNp63α is present in cancer cells, survival will be induced. In contrast, when p63 is not present, homodimers of TAp73 will be formed and will promote pro-apoptotic gene expression (Puma and Noxa for instance). In HNSCC, both ΔNp63α and TAp73 are expressed but TID of ΔNp63α is capable of inhibiting pro-apoptotic function of the TA isoform, thus promoting survival and cancer progression.

HNSCC presents mutated p53, as it is in the majority of cancers, with mutations that are found in more than half of HNSCC malignancies. Rather than being mutated, p73 is overexpressed in a wide range of tumor types, including breast, lung, colon, and stomach cancers, as well as epithelial cancers such as HNSCC, with TAp73 being the most common isoform [22]. The P63 gene region (chromosome 3q27-28) is frequently amplified in squamous cell carcinomas [23,24] and its consequent protein overexpression is seen in up to 80% of HNSCCs. In these types of cancers, Np63 is the most prevalent isoform. ΔNp63α is the most predominant isoform in these kinds of malignancies [25,26]. As both partners possess homologous ODs, they are susceptible to interact and form homo- and hetero-oligomers (Figure 2). When p63 is expressed in cancer cells, homodimers between two ΔNp63α molecules (the most common expressed isoform) will be formed and will repress some apoptotic promoters as Puma and Noxa, promoting survival. In contrast, when p63 is absent, TAp73 is the most usual isoform which will form dimers as well, but this time promoting the expression of apoptotic genes.

In the case where both populations coexist, heterodimers between ΔNp63α and TAp73 can be formed and are crucial for tumor maintenance. There is a balance that is established between both isoforms that can be destabilized if one of the isoforms surpasses the other. Depending on whether the isoform is more frequent, this disturbance of balance might result in improved survival or apoptotic characteristics. Physical interactions between p63 and p73 have been shown to be significantly stronger than homodimers, which is interesting. The main isoform in the HNSCC environment is ΔNp63, which results in Tap73-Np63 heterodimers. The TID of the dominant protein inhibits the TA isoform’s transcriptional activity, resulting in a survival phenotype.

The above-described studies emphasize the fact that HNSCC is a good model to better understand the involvement of p63 and p73 in cancer, thanks to its inactive p53. Several teams have used it to assess the implications of p63 (ΔNp63α) and p73 (TAp73β) in the cancer metastatic dissemination, in particular through the regulation of miRNA networks [27,28,29].

## 3. p53 Protein Family in Primary Tumors and Metastatic Dissemination

Mutated p53 itself has been shown to drive invasion by promoting [30], sometimes through integrin recycling. Indeed, the Vousden and Muller teams demonstrated that mutants p53 can induce a loss of directionality of migration, promote invasion, and interfere with the metastatic behavior [30,31]. The functions of p63 and p73 in the cancer context have been deeply studied [8,27,32,33]. Regarding p73, several knockdown (KD) in vivo experiments were done to evaluate the role of both TAp73 and ΔNp73 isoforms. An increased susceptibility to spontaneous tumor formation was observed in a TAp73 KD model of lung adenocarcinoma [34,35]. In contrast, the overexpression of ΔNp73 inhibited spontaneous senescence and permitted primary fibroblast transformation in cooperation with Ras protein [34,35]. This overexpression has also been shown to induce fibrosarcomas in vivo. These data give evidence of the role of TAp73 in tumor suppression, whereas ΔNp73 acts as a tumor promoter.

It has been reported that the p63 protein is associated with metastasis regulation. The TAp63 isoform was described as a metastasis suppressor by decreasing cell motility and invasion [36], and the ΔNp63 isoform has been described as a pro-metastatic protein through the regulation of the *brachyury* gene, an essential gene for limb development which is also involved in EMT in several tumor cell lines (stomach, ovary, prostate, and several others). Indeed, ΔNp63 was described as a promoter of cell proliferation, migration, and invasion [37,38]. Finally, recent contradictory studies describe the ΔNp63 isoform as a key player in EMT; on one hand as a pro-EMT effector in keratinocytes and on the other hand as an anti-EMT effector in breast cells, both activities in a TGFβ-dependent manner [39,40]. The Melino teams demonstrated that p63 might inhibit metastasis by its ability to interact with p53 [41], and specifically in prostate cancer through the regulation of miR-205; this miR being essential for the inhibitory effects of p63 on markers of the EMT [42]. Flores also demonstrated that the Tap63 isoform was suppressing metastasis through both the regulation of Dicer and miRNAs 130b [36]. Another team demonstrated that ΔNp63 was contributing to the inhibition of HER2-induced metastasis [43].

Despite the disagreement encountered in the literature, all this evidence makes p63 a new and interesting player in the metastasis phenomenon.

### p53 Protein Family and microRNA-Regulated Metastasis

Numerous studies have now demonstrated that miRNAs can play key roles in both oncogene and tumor suppressor pathways. An emerging consensus is that miRNAs are particularly prominent within regulatory circuits controlling transcription factor functions, as it happens in the p53 protein family. Although we are only beginning to uncover their complexity, such circuits may be particularly important within the regulation of metastasis [27,32,44,45,46].

**Figure 3:**
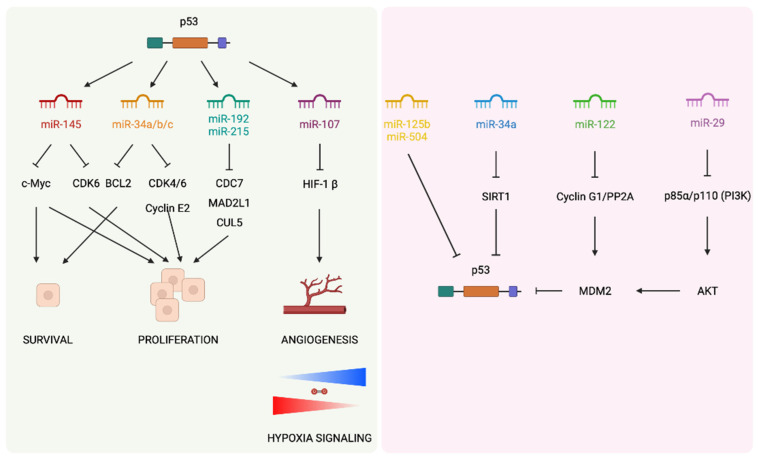
p53 regulates the expression of several miRNAs for further regulation of various processes of the metastatic dissemination (**left side**). microRNAs might also be negative regulators of p53 (**right side**).

Some evidence has shown how p53 regulates the metastatic dissemination through miRNAs. One example of it is the direct transactivation of miR-34 family expression. miR-34 is a family of oncosuppressor miRNAs that is usually downregulated in several tumors such as neuroblastoma, lung, or pancreatic cancers [47]. The ectopic expression of miR-34 inhibits proliferation, EMT, migration, invasion, and metastasis both in vitro and in vivo through interference with cell cycle arrest, apoptosis, and cell senescence pathways [48]. Moreover, p53 controls EMT by the regulation of other miRNAs; induced expression of miR15a/16-1 suppresses lung metastatic colonization in a xenograft model [49]. MicroRNA-145 is also upregulated by p53 to modulate EMT and stemness properties in prostate cancer cells; the suppression of migration, invasion, EMT, and cancer stem markers are observed when miR-145 is expressed. The loss of p53 may promote bone metastasis at least partially through repressing miR-145 [50]. Additionally, p53 miRNA-dependent regulation can be bidirectional. Several studies demonstrated that miRNAs can also regulate p53 expression, adding even more complexity to the regulatory systems that drive the metastatic dissemination (Figure 3) [51].

Unlike p53, the contribution of miRNAs to p63 and p73 functions is not so clearly defined but, similar to p53, they may be potentially involved within the metastatic dissemination process. Recent studies have now begun to shed light on the critical downstream transcriptional target genes (including microRNAs) and functions of p63 [52,53,54]. One study showed that miR-193a-5p is transcriptionally repressed by p63 and activated by p73 in a squamous cell carcinoma model [32]. A p63/p73 crosstalk in which miR-193-5p is involved may explain chemoresistance to treatment. After treatment with cisplatin, p63 is degraded whereas p73 is activated. This promotes the expression of miR-193-5p, which in turn represses pro-apoptotic protein p73, making cancer cells resistant to the treatment. In that way, the inhibition of miR-193-5p could be considered as a therapeutic option in order to increase the p73-dependent chemosensitivity [8,32]. Recently, Flores’s team observed that the oscillatory expression of ΔNp63 was crucial for metastatic dissemination in breast cancer. Moreover, they demonstrated that this regulation was under the supervision of miRNAs [55]. Very interestingly for the present review, they established a connection with the TGFβ pathway observing a TGFβ-regulated miRNA network acted as upstream regulators of this oscillatory expression of ΔNp63 during cancer progression.

## 4. The TGFβ Pathway

**Figure 4:**
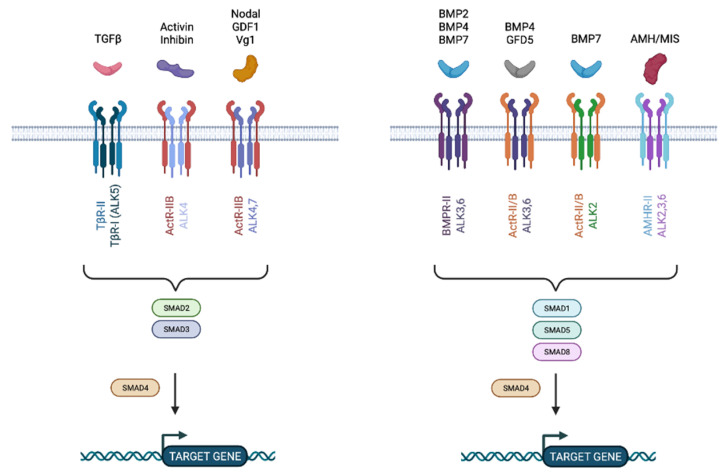
Schema of all the described TGFβ ligands and Type I and II receptors.

The TGF pathway is one well-known pathway that has been linked to metastatic spread. TGFs are a class of structurally related polypeptide growth factors that regulate a wide range of cellular activities such as cell proliferation, lineage determination, differentiation, motility, adhesion, and cell death, making them important players in development, homeostasis, wound repair, and cancer. TGF, activins and inhibins, bone morphogenetic proteins (BMPs), and Müllerian inhibiting substance (MIS) are all members of this family (Figure 4) [56]. TGFβ factors are active as dimers that are stabilized by hydrophobic interactions; notably by disulfide bonds between cysteine residues [57].

TGFβ factors exert their effects through binding to specific cell surface receptors, classified into two subfamilies based on their structural and functional properties; Type I and Type II receptors. There are several Type I receptors; TGFβ receptor (TβRI), activin receptor (ActR-IB), and two BMP receptors (BMPIR-IA and IB). The Type II receptor subfamily includes TβR-II, BMPR-II, and AMHR, which selectively bind TGFβ, BMPs, and MIS, respectively (Figure 4). Those receptors are composed of a cysteine-rich extracellular domain allowing their ligands to bind, a transmembrane region, and a kinase domain, where the serine/threonine domain is found. Type II receptors typically contain a short C-terminal extension after the kinase domain which is absent in Type I receptors. In contrast, Type I receptors contain a characteristic extension GSGSG sequence, termed the GS domain. The activation of Type I receptors relies on the phosphorylation of its GS domain by the Type II receptors, generating a complex that phosphorylates and recruits downstream pathway effectors.

In the case of TGFβ, three different isoforms, TGFβ1, TGFβ2, and TGFβ3, exhibit similar properties. TGFβ initiates signaling by binding to the Type II receptor which will permit the phosphorylation and recruitment of the Type I receptor, forming a heteromeric complex of Type I and II receptors. The signal will then propagate through phosphorylation of the SMAD proteins.

**Figure 5:**
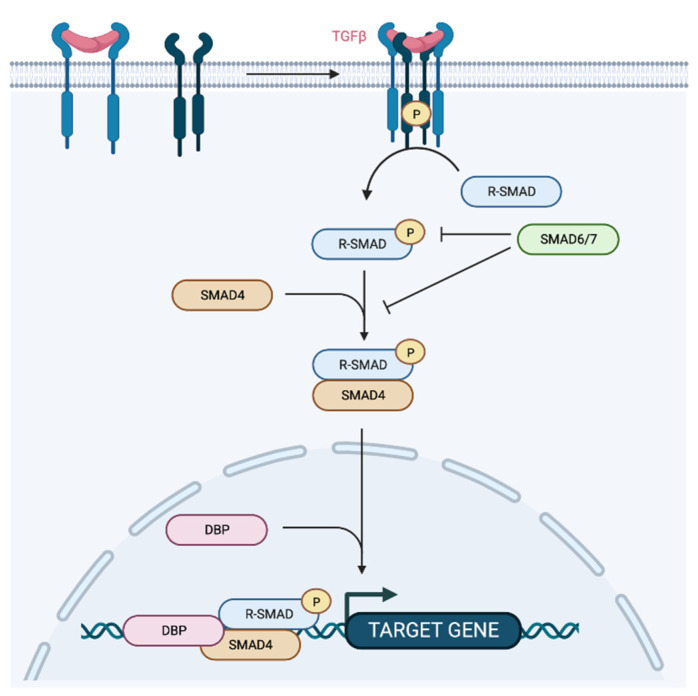
TGFβ pathway. Binding of TGFβ to TβRII and recruitment of TβRI leads to the formation of a receptor complex and the phosphorylation of TβRI. Further phosphorylation of receptor-regulator SMADs (R-SMADs) will allow recruitment and association to SMAD4 and translocation into the nucleus to associate with DNA-binding partners to activate target gene transcription. SMAD6 and SMAD7 interfere with the TGFβ pathway.

There are eight different SMAD proteins, classified in three groups: (1) the receptor-regulated SMADs (R-SMAD), the co-mediator SMAD (Co-SMAD), and the inhibitory SMAD (I-SMAD). R-SMADs (SMAD 1, 2, 3, 5c and 8) are directly phosphorylated and activated by Type I receptor kinases and form heteromeric complexes with Co-SMAD (SMAD4) for further translocation into the nucleus to regulate the transcription of target genes (Figure 5). I-MADs, SMAD6 and SMAD7, negatively regulate TGFβ signaling by competition with R-SMADs for receptor, Co-SMAD interaction or by targeting the receptors for degradation [58]. Once translocated into the nucleus, SMAD complexes recruit transcriptional coactivators, corepressors, and chromatin remodeling factors permitting the activation or repression of hundreds of target genes at once [59].

### 4.1. TGFβ Pathway in Cancer

#### 4.1.1. TGFβ as a Tumor Suppressor

In normal conditions, TGFβ is a ubiquitously expressed cytokine that, in addition to its role in cell development, differentiation, and survival regulation, also inhibits the proliferation of epithelial, endothelial, and hematopoietic cell lineages [59]. Indeed, there is evidence that shows the role of TGFβ as tumor protector. Transgenic mice overexpressing TGFβ in their mammary glands showed cell hypo-proliferation, poor mammary duct development, as well as no spontaneous breast tumor formation [60]. TGFβ maintains tissue homeostasis and prevents incipient tumors from progressing by also regulating the cellular microenvironment; as observed in mice with SMAD4-deleted T-cells which develop gastrointestinal tumors with higher incidence [61]. Moreover, TGFβ production by tumor-infiltrating lymphocytes strongly suppresses tumor growth in colon cancer through the inhibition of the cytokine IL-6, implicated in chronic inflammation and carcinogenesis [62].

TGFβ-tumor protector effects have been explained through a reduction in *c-Myc* levels [63] and the concomitant stimulation of some cyclin-dependent kinase inhibitors (mainly p15^INK4B^ and p21^CIP1^ involved in cell cycle [64]). Moreover, the SMAD-dependent TGFβ pathway has also been implicated in tumor suppression by acting upstream of the cyclin-dependent kinase inhibitors and c-Myc. Genetic lesions in key effectors of the pathway have also been described: TGFβR2 mutations are found in 20–25% of colorectal cancers [65] whereas mutations in SMAD2 and SMAD4 were also observed in colorectal and pancreatic carcinomas [66,67]. Transgenic expression of a dominant negative TGFβR2 enhanced the incidence of mammary tumors after stimulation with 7,12-dimethylbenz-[a]-anthracene carcinogen agent [68]. Similarly, the expression of TGFβR2 in colon or breast carcinomas showed growth inhibition, suppression of anchorage independence, and reduced tumor formation and metastasis in vivo [69] whereas dominant negative TGFβR2 abolished the ability of TGFβ to inhibit cell growth, to promote cell differentiation, and to induce apoptosis [70].

#### 4.1.2. TGFβ as a Tumor and Metastasis Promoter

Despite all the evidence showing its tumor suppressor role, resistance to TGFβ-mediated cytostasis is a hallmark of neoplastic transformation. Cancer cells have the capacity to avoid or to adulterate the suppressive influence of the TGFβ pathway, either through the inactivation of principal components of the pathway (by mutation or other mechanisms) or by downstream alterations that disable just the tumor-suppressive arm of this pathway.

Abundant amounts of TGFβ are usually present at tumor lesions, initially preventing premalignant progression but eventually as a factor that cancer cells use to their own advantage. TGFβ can be secreted by cancer cells themselves but also by the tumor stroma; the presence of tumor-infiltrating cells coincide with TGFβ secretion [59]. This conversion in the TGFβ function is known as the “TGFβ paradox” [71] and underlies the adverse prognosis that is associated with tumor growth, epithelial to mesenchymal transition (EMT) and invasion, evasion of immune surveillance, cancer cell dissemination and metastasis, as well as chemoresistance development. It is interesting to note that, depending on the cell type and the environment, the cellular response to TGF-beta might vary greatly. TGF-beta can induce epithelial-mesenchymal transition and mediate fibroblast activation, responses that are implicated in promoting carcinogenesis, and fibrotic diseases, whereas it can also cause epithelial cells to undergo growth arrest and apoptosis, responses which are crucial for suppressing carcinogenesis [59,72]. The molecular mechanisms whereby TGFβ promotes the progression of late stage carcinomas as well as cancer cell invasion and metastasis have not been fully elucidated. Collectively, recent findings establish a paradigm whereby TGFβ potently inhibits tumor initiation and the development of early-stage carcinomas but enthusiastically drives the metastatic progression of late-stage carcinomas [73].

TGFβ was shown to induce EMT in breast cancer, squamous carcinoma, ovarian adenosarcoma, and melanoma. Mammary epithelial cells underwent EMT in the presence of TGβ after transformation by the Ras oncogene [74]. The cytoskeleton reorganization that was mediated by TGFβ, mainly based in the downregulation of E-cadherin and β-catenin, ended up in the spindle shape acquisition, the so-called mesenchymal phenotype and increased motility and scattering potentials by cancer cells. A crosstalk of the TGFβ pathway with MAPKK and Pi3K pathways were also reported to be important in the acquisition of the EMT and invasive features [75]. High TGFβ expression within the tumor microenvironment was also reported as important in the induction of MMP-2 and MMP-9 proteins in both tumor and endothelial cells, promoting invasion and angiogenesis.

TGFβ signaling was also shown to promote breast cancer metastasis in lung and bones. Recent evidence showed that enforced TGFβ signaling inhibited mammary tumor formation whereas it facilitated extravasation of Neu-induced cells to form lung metastasis. Despite the tumor suppressive effects at the primary site, TGFβ enhanced metastasis occurrence [76]. Furthermore, the apparition of the TGFβ-mediated lung metastasis was explained by the induction of angiopoietin-like 4 (ANGPTL4) via SMAD signaling. ANGPTL4 expression was responsible for enhancing the disruption of endothelial cell–cell junctions and transendothelial passage for further retention in the lungs [77].

In the case of bone osteolytic metastasis, it has been proposed that TGFβ released from bone matrix resorption stimulates tumor cells to produce PTHrP and IL-11, both promoting tumor growth and exacerbating osteolysis [78]. PTHrP and IL-11 are osteolysis-promoter factors that are released by cancer cells upon TGFβ stimulation through the crosstalk of the TGFβ pathway with p38 MAP kinase pathway [79]. Breast cancer bone metastases show active SMAD signaling, proven by the accumulation of phosphorylated SMAD2 in the nucleus of tumor cells. The knockdown of SMAD3 in breast cancer cells in vivo resulted in retarded growth of bone metastasis [80]. These data highlight the importance that both dependent and independent roles that SMAD signaling plays in the pro-metastatic role of TGFβ.

Moreover, the crucial involvement of the TGF-β/SMAD3 signaling pathway in YAP-driven lung metastasis development in Osteosarcoma (OS) has recently been highlighted. Overexpression of mutant versions of YAP able or not to interact with TEAD was used to investigate the molecular processes by which YAP governs metastasis development. RNA-sequencing analysis and gene set enrichment were used to find molecular signatures. The proximity ligation assay (PLA), immunoprecipitation, and promoter/specific gene assays were used to investigate the interactions between YAP and SMAD3. The role of the TGF-pathway in the ability of YAP to induce metastatic development in vivo was investigated using a TGF cascade inhibitor in a preclinical model of OS and in vitro on the migration and invasion ability of OS cells [81]. As perfectly illustrated by the studies that are described above [55], the TGFβ pathway is somehow connected to the p53 protein family, either regulated by some of its members or the inverse, and the TGFβ pathway might regulate the expression of the p53 family proteins. This highlights the potential role of the p53 family-TGFβ axis during the metastatic process.

## 5. Role of p53 Protein Family in TGFβ Pathway and Metastasis

Several teams have already investigated the potential interplay between the p53 protein family and the TGFβ pathway in the control of the metastatic process. The Yong Yi team demonstrated that TGFβ1 was promoting migration and metastatic dissemination through the induction of Tap63α lysosomal degradation in a pancreatic cancer model [82]. Even more recently, the noncanonical TGFβ signaling has been observed to promote breast cancer metastasis through the FBXO3-mediated degradation of ΔNp63α [83].

TGFβ1 has been shown to be activated indirectly by p73. Indeed, the Huang team observed that lncRNA TP73-AS1 axis was repressing the miR-539 expression, promoting MMP-8 expression, and activating TGFβ1 signaling to induce M2 macrophage polarization in hepatocellular carcinoma [43]. On the other hand, other teams demonstrated an opposite effect in pancreatic ductal adenocarcinoma. The Tomasini team showed that Tap73 loss led to the activation of TGFβ signaling through a SMAD-independent pathway, stimulating the EMT process [84]. Tap73alpha was observed in gastric cancer to bind to the promoters of Bax and Puma and mediate TGFβ-induced apoptosis [85].

In coherence with this finding and the knowledge regarding the different p73 isoforms, Coppes et al. observed that ΔNp73 enhances promoter activity of TGFβ-induced genes and stimulates the expression of TGFβ signaling targets [86].

The TGFβ pathway can obviously control p53 family protein members, but reciprocally the TGFβ pathway can be modulated by the p53 family. For instance, p53 miRNA-dependent mechanisms are strongly involved in the metastatic dissemination through their interaction with the TGFβ pathway. From one hand, p53 induces the oncosuppressor miR-127 whereas the presence of TGFβ decreases its expression through the upregulation of the miR-127-inhibitory c-Jun. A feedback regulation is established between miR-127 and the TGFβ/c-Jun cascade in hepatocellular carcinoma involving a crosstalk between the oncogene c-Jun and tumor suppressor p53. Low levels of miR-127 correlate with high MMP13 expression and higher invasion potential [87]. Moreover, ΔNp63α has been shown to control the TGFβ pro-metastatic potential through the regulation of several microRNAs [27].

Collectively, these findings define a link between p53, miRNA expression, and epithelial plasticity that can be potentially used by cancer cells at any step of metastatic tumor progression [88].

Moreover, other evidence describes a binding between p63 and mutant p53 to regulate the TGFβ-induced metastasis. The TGFβ switch towards metastatic phenotypes can be acquired by the combined action of two common oncogenic lesions, Ras and p53-mutation. Mutant-p53 and SMADs form complexes which intercept p63 to form a ternary complex in which p63 would be responsible for TGFβ malignant effects being unleashed. In this case, the inactivation of p63 transforms normal cells into malignant tumors rescuing metastatic ability [89].

## 6. Conclusions

The p53 protein family is unanimously associated with the metastatic dissemination in various cancers, but depending on the cells involved, on the isoforms and on the context, it can be associated with an inhibitory role or an inducing role [27,41,55]. MicroRNAs appear to be frequently involved at various levels in this regulatory process [32,45], sometimes upstream and sometimes downstream of the p63 and/or p73 proteins [27,55]. The numerous different isoforms for p63 and p73 that are associated with the TGFβ implication in this process adds even more complexity because of its well-known dual effect, and this is probably one of the reasons why we observe so many discrepancies in the literature regarding the pro- or anti-metastatic effect of p63 and p73. Indeed, similar to the roles of the miRNAs, the role of the TGFβ pathway is sometimes upstream [90] and sometimes downstream of p63 or p73 [27].

A better understanding of the epigenetic regulation of the p53 protein family transcription might be one of the key comprehensions, in particular the chromatin and the enhancers regulation that are highly cell- and context-dependent, such as the metastasis-dependent p53 family-miRNA-TGFβ axis.
